# Acupressure Versus Ondansetron Usage for Postoperative Nausea and Vomiting After Gynecologic Surgeries

**DOI:** 10.7759/cureus.36862

**Published:** 2023-03-29

**Authors:** Elif Ongel, Ezgi Erdag, Esra Adiyeke, Nurten Bakan

**Affiliations:** 1 Anesthesiology and Reanimation, Sancaktepe Martyr Prof. Dr. Ilhan Varank Education and Research Hospital, Istanbul, TUR

**Keywords:** postoperative nausea and vomiting, gynecologic surgical procedures, therapeutics, ondansetron, acupressure

## Abstract

Introduction

Anti-emetic interventions include pharmacologic and non-pharmacologic strategies. Acupressure is a non-pharmacologic and non-invasive therapeutic method that involves applying physical pressure to acupuncture points with fingers or devices. The pericardium (PC6) acupoint is located on the palm side of the wrist between the palmaris longus and flexor carpi radialis tendons, three fingers across the wrist starting at the wrist crease. Our first aim was to assess the effect of PC6 point acupressure on PONV after gynecological surgeries compared to intravenous (IV) ondansetron. Secondly, we aimed to assess the factors associated with the first^ ^and second hours (early) postoperative nausea scores.

Methods

This was a prospective, randomized, and single-centered intervention study conducted between November 1, 2022, and December 31, 2022, in a tertiary care hospital. Sancaktepe Martyr Prof. Dr. Ilhan Varank Education and Research Hospital Ethical Committee provided ethical approval for this study on October 14, 2022 (No: E-46059653-020). Randomization was done using the lottery method. Patients, who were over the age of 18 with an American Society of Anesthesiologists (ASA) physical score of I, II, or III status and had undergone gynecologic surgery under general anesthesia, were included. Patients, who were ASA IV, under continuous use of opioids or corticoids, underwent surgery with regional anesthesia, or declined to participate in the study, were excluded. There were two comparisons in this study. First, we divided patients into two groups according to anti-emetic prevention. Patients, who received IV 4 mg ondansetron (Group O), and patients, who placed acupressure bands at the P6 points on both forearms (Group B). The second comparison was done to assess the factors associated with early postoperative nausea. Patients were divided into two groups according to the mean early postoperative nausea scores as low (< 4, Group 1) and high/moderate (≥ 4, Group 2). PONV and pain scores were collected at five-time points: the first, second, sixth, twelfth, and twenty-fourth hours after surgery.

Results

Of 102 patients, 50 were in Group O and 52 were in Group B. There was no significant difference in postoperative pain, nausea, and vomiting scores. Fifty patients (50%), including 24 patients (48%) in Group O and 26 patients (52%) in Group B, experienced early moderate/high postoperative nausea in our study. According to the second comparison, 52 patients were in Group 1, and 50 patients were in Group 2. Operation time; first and second-hour pain scores; first, second, sixth, twelfth, and twenty-fourth-hour scores; and first and second-hour vomiting scores were all significantly different across groups.

Conclusion

The effect of PC6 point acupressure on early PONV compared to IV ondansetron was similar after gynecological surgeries. However, using only one anti-emetic treatment did not adequately relieve early PONV. Of all patients, 11 (10%) required an extra anti-emetic medication at the ward. 50% of patients experienced early moderate/high postoperative nausea in our study. Motion sickness history, operation time, and early postoperative pain scores were associated with early PONV.

## Introduction

Postoperative nausea and vomiting (PONV) is defined as any nausea or vomiting that occurs during the postoperative 24-48 hours [1]. PONV, which needs to be treated, is an important and uncomfortable symptom. The incidence of PONV was reported to be 30% in all postoperative patients and 80% in high-risk patients [2]. Dehydration, aspiration, infection, electrolyte imbalance, and prolonged hospital stay, which may increase treatment costs, were found as the complications of PONV [3].

Assessing the pathophysiology and risk factors of PONV can provide a more rational approach to prevention and treatment [2]. The pathophysiology of nausea is a complex mechanism, that is associated with psychological states, and multiple systems, including the autonomic nervous, central nervous, and endocrine [4]. Using multimodal anti-emetic treatments with different sites of action might reduce the incidence of PONV. However, anti-emetic drugs may cause some side effects like sedation, fatigue, constipation, and headache [5]. Hence, anesthetists are looking for some inexpensive and non-invasive methods to prevent and treat PONV. PONV can be managed by complementary strategies, like acupressure.

Acupressure is a non-invasive therapeutic method that involves applying physical pressure to acupuncture points with fingers or devices [6,7]. The popularity of acupressure has increased over decades, and acupressure studies have focused on the 'pericardium (PC6) acupoint' to manage nausea and vomiting. PC6 acupoint has been among the five preferred complementary and alternative therapies in hospitalized patients [8]. PC6 is located on the palm side of the wrist between the palmaris longus and flexor carpi radialis tendons, three fingers across the wrist starting at the wrist crease.

Risk factors for PONV might be related to the patient, surgery, or anesthesia. Due to this multifactorial situation, it is unlikely to show definite causes and treatments of PONV. Apfel et al. described the independent risk factors for PONV, including female gender, history of PONV or propensity for motion sickness, non-smoking, and intraoperative opioid use [1]. The last guideline presented risk factors as female sex, younger age, non-smoker, surgery type, history of PONV/motion sickness, and opioid analgesia. They recommended using anti-emetic prophylaxis, including ondansetron and acupressure [9]. Our study group had a moderate risk for PONV, including at least two risk factors; female gender and opioid use, and we applied anti-emetic prophylaxis to all patients according to the guideline recommendation.

Previous studies of PC6 point acupressure for managing PONV described conflicting results. Therefore, our first aim was to assess the effect of PC6 point acupressure on PONV after gynecological surgeries compared to intravenous (IV) ondansetron. Secondly, we aimed to assess the factors associated with early PONV, which occurs within four hours after surgery [10].

## Materials and methods

Study design

This study was a prospective, randomized, and single-centered intervention study conducted between November 1, 2022, and December 31, 2022, in a tertiary care hospital. Randomization was done using the lottery method. Sancaktepe Martyr Prof. Dr. Ilhan Varank Education and Research Hospital Ethical Committee provided ethical approval for this study on October 14, 2022 (No: E-46059653-020).

Identification of study participants

Patients over the age of 18 with an American Society of Anesthesiology (ASA) physical status I, II, or III, who were scheduled to undergo elective gynecologic surgery under general anesthesia assisted by the same anesthesiology specialist were considered for enrollment in this study. Patients who were ASA IV, under continuous use of opioids or corticoids, underwent surgery with regional anesthesia, or declined to participate in the study, were excluded. Patients' consent for study participation was obtained from them before the surgery. Power analysis was run to evaluate the adequate size of the sample. To obtain a statistical power of 80% with an effect size of 0.41 in the study, we needed to enroll a minimum of 2 × 46 subjects to detect significant differences between groups.

Data collection

All patients were hospitalized on the morning of surgery. Patient's age, weight (kg), height (cm), smoking status, ASA scores, previous postoperative nausea and vomiting history, motion sickness, comorbidities (hypertension, diabetes mellitus, asthma, thyroid disease), and Apfel scores were recorded. Patients were asked to complete the Hospital and Depression Scale (HADS) before surgery [11].

After standard monitorization (electrocardiography, non-invasive blood pressure, and pulse oximeter) of all patients, premedication with IV midazolam (0.03 mg/kg) was applied. General anesthesia was induced with IV propofol (2 mg/kg), fentanyl (2 μg/kg), and rocuronium (0.6 mg/kg) and maintained with sevoflurane in a mixture of 50% oxygen and 50% air with a 2 L/dk flow rate. Intraoperatively, analgesia was provided with remifentanil infusion (0.05 to 0.25 μg/kg/min) titrated to maintain heart rate and blood pressure within ± 20% of the baseline values. The lungs were ventilated with a tidal volume of 6-8 mL/kg and a positive end-expiratory pressure of 5 cmH_2_O. End-tidal carbon dioxide was maintained at between 35 and 40 mmHg by adjusting the respiratory rate. Intraoperative administered fluid was calculated according to fasting hours, the patient’s weight, and the type of surgery. Diuresis, first and last heart rates and mean artery pressures, type of surgery, and operation time were recorded. Intraoperative administered analgesics for postoperative pain included intravenous paracetamol (1000 mg) and morphine (0.1 mg/kg). Intraoperative administered anti-emetics for PONV were IV 4 mg ondansetron or P6 acupressure bands. If patients who received intraoperative ondansetron, would feel nausea at the ward, they were planned to treat with IV 10 mg metoclopramide. If patients who have placed acupressure bands, would feel nausea in the ward, they were planned to treat with IV 4 mg ondansetron.

There were two comparisons in this study. First, we randomly divided patients into two groups according to anti-emetic treatment. Patients who received IV 4 mg ondansetron (Group O), and patients who placed acupressure bands on the P6 points of both forearms (Group B). Ondansetron or an acupressure band were applied with analgesics 30 minutes before the end of the surgery. The bands were elastic and came in three sizes; small, medium, and large. The appropriately sized band was applied to each patient according to the patient’s wrist width by the same anesthesiology specialist. The patient’s palm and fingers were examined for any signs of extreme compression due to the acupressure band.

Patients were asked to record their acute pain, nausea, and vomiting with numerical rating scores (NRS) at the first, second, sixth, twelfth, and twenty-fourth hours after surgery on the given sheets. Sheets and bands were collected 24 hours after surgery. Hospital length of stay (LOS) days were recorded after patients' discharge.

The second comparison was done after completing the study to assess the factors associated with early PONV. The patients were divided into two groups according to the mean of postoperative first and second-hour nausea scores as low (< 4, Group 1) and high/moderate (≥ 4, Group 2).

Data analysis

The mean, standard deviation, median, minimum, maximum value frequency, and percentage were used for descriptive statistics. The distribution of variables was checked with the Kolmogorov-Simirnov test. The independent samples t-test and Mann-Whitney U test were used for the comparison of quantitative data. The Wilcoxon test was used for the repeated measurement analysis. The chi-square test was used for the comparison of qualitative data. IBM SPSS Statistics for Windows, Version 28.0 (Released 2021; IBM Corp., Armonk, New York, United States) was used for statistical analyses.

## Results

**Figure 1:**
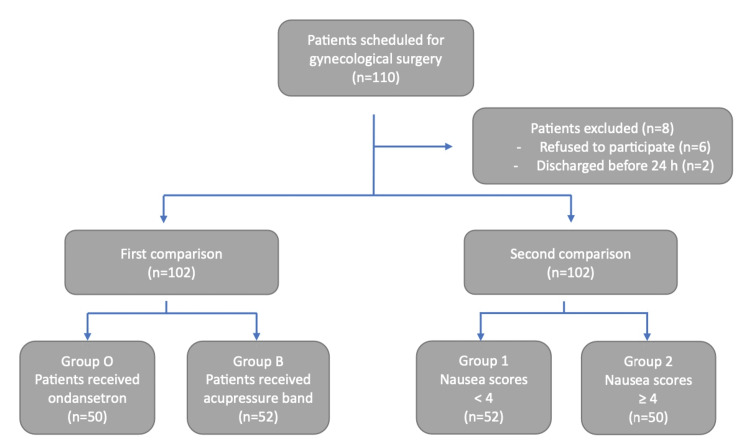
Flow chart

One hundred and ten patients, who had undergone elective gynecological surgeries between November 1, 2022, and December 31, 2022, were considered suitable for our study. Six patients declined to participate in the study, and two patients were discharged before 24 hours with the sheets. Participants were 102 patients, aged between 19 and 79, and underwent gynecological surgeries assisted by the same anesthesiologist (Figure 1).

There were two comparisons in our study. The first comparison divided patients into two groups according to anti-emetic interventions: ondansetron (Group O) and acupressure bands (Group B). There was no significant difference between groups in demographic variables. Perioperative variables included operation type, administered fluid, diuresis, hemodynamics of patients, operation time, and hospital LOS. Intraoperative administered crystalloid fluid (p=0.005), heart rate after surgery (p=0.004), heart rate variation (p=0.045), operation time (p=0.001), and hospital LOS (p=0.013) were significantly different between groups. There was no significant difference in postoperative pain, nausea, and vomiting scores (Table 1). Twenty-four patients (48%) in Group O and 26 patients (52%) in Group B had moderate/high early PONV scores (p=0,840). No side effects or complications were observed due to the acupressure bands. Ten patients (19%) reported removing and placing back the acupressure bands because they felt uncomfortable.

**Table 1:** Demographic and perioperative variables of Group O and Group B Data are presented as the mean ± SD, absolute number (percentage), or median (interquartile range). *, Mann-Whitney U test; ^†^, independent sample t-test; ^§^, chi-square test P values in bold represent statistically significant results (P < 0.05). BMI: Body mass index; ASA: American Society of Anesthesiologists; PONV: Postoperative nausea and vomiting; HADS: Hospital anxiety and depression scale; LOS: Length of stay

Variables	Total (n= 102)	Group O (n= 50)	Group B (n= 52)	P value
Age, year	45 ± 10	45 ± 12	45 ± 9	0.527*
Height, cm	161 ± 6	161 ± 6	161 ± 6	0.495*
Weight, kg	73 ± 12	73 ± 13	74 ± 12	0.768^†^
BMI	28 ± 7	29 ± 10	28 ± 5	0.965*
Non-smokers	73 (71%)	39 (78%)	34 (65%)	0.158^§^
ASA scores, n				0.968^§^
I	11 (11%)	5 (10%)	6 (11%)	
II	81 (79%)	40 (80%)	41 (79%)	
III	10 (10%)	5 (10)	5 (10)	
Apfel scores, n				0.408^§^
II	23 (22%)	9 (18%)	14 (27%)	
III	57 (56%)	28 (56%)	29 (56%)	
IV	22 (22%)	13 (26%)	9 (17%)	
Previous PONV, n	15 (15%)	7 (14%)	8 (15%)	0.724^§^
Motion sickness, n	28 (28%)	15 (30%)	13 (25%)	0.572^§^
Comorbidities, n				
HT	19 (19%)	8 (16%)	11 (21%)	0.504^§^
DM	13 (13%)	7 (14%)	6 (12%)	0.709^§^
Asthma	8 (8%)	2 (4%)	6 (12%)	0.157^§^
Thyroid disease	7 (7%)	4 (8%)	3 (6%)	0.656^§^
HADS				
Anxiety	7 ± 4	7 ± 4	7 ± 5	0.670*
Depression	6 ± 6	5 ± 3	6 ± 3	0.135*
Operation type, n				0.068^§^
Laparoscopy	29 (28%)	11 (22%)	18 (35%)	
Laparotomy	41 (40%)	18 (36%)	23 (44%)	
Vaginal	32 (32%)	21 (42%)	11 (21%)	
Intraoperative crystalloid fluid, mL	1250 (975- 2000)	1110 (775- 1600)	1600 (1000-2000)	0.005*
Diuresis, mL	300 (200- 500)	350 (200- 600)	250 (200- 500)	0.164*
Heart rate, per min				
Before surgery (First recorded)	86 ± 15	85 ± 14	86 ± 15	0.667^†^
After surgery (Last recorded)	84 ± 14	80 ± 15	88 ± 13	0.004^†^
Variation	-2 ± 16	-5 ± 19	2 ± 15	0.045^†^
Mean artery pressure, mmHg				
Before surgery (First recorded)	96 ± 13	93 ± 10	96 ± 13	0.569*
After surgery (Last recorded)	92 ± 14	89 ± 12	94 ± 14	0.063*
Variation	-4 ± 14	-4 ± 12	-2 ± 15	0.330*
Operation time, min	115 (75-160)	89 (57- 122)	137 (86- 178)	0.001*
Postoperative pain scores				
1^st^ hour	7.4 ± 2.9	7.4 ± 3.1	7.5 ± 2.7	0.945*
2^nd^ hour	6.3 ± 2.9	6.1 ± 3	6.5 ± 2.8	0.495*
6^th^ hour	5.2 ± 2.9	4.9 ± 2.8	5.4 ± 3	0.355*
12^th^ hour	3.8 ± 3.1	3.5 ± 3.1	4.1 ± 3	0.332*
24^th^ hour	2.9 ± 3	2.7 ± 2.8	3.1 ± 3.1	0.438*
Postoperative nausea scores				
1^st^ hour	3.6 ± 3.9	3.5 ± 4	3.6 ± 3.9	0.888*
2^nd^ hour	3.5 ± 4.1	3.7 ± 4.3	3.3 ± 3.9	0.608*
6^th^ hour	2.6 ± 3.8	2.7 ± 3.9	2.6 ± 3.7	0.850*
12^th^ hour	1 ± 2.6	1.1 ± 2.7	0.9 ± 2.4	0.679*
24^th^ hour	0.3 ± 1.3	0.1 ± 0.5	0.5 ± 1.8	0.096*
Postoperative vomiting scores				
1^st^ hour	2.4 ± 3.9	1.8 ± 3.5	2.9 ± 4.3	0.325*
2^nd^ hour	2.2 ± 3.9	2.1 ± 3.9	2.3 ± 3.9	0.647*
6^th^ hour	1.9 ± 3.8	1.7 ± 3.7	2.6 ± 3.7	0.569*
12^th^ hour	0.6 ± 2.2	0.8 ± 2.5	0.9 ± 2.4	0.434*
24^th^ hour	0.1 ± 1	0 ± 0	0,5 ± 1,8	0.163*
Anti-emetic treatment at ward, n	11 (11%)	5 (10%)	6 (11%)	0.802^§^
Hospital LOS, day	2.7 ± 0.9	2.5 ± 0.9	2.8 ± 0.9	0.013*

The second comparison divided patients into two groups according to the mean early postoperative nausea scores: low nausea scores < 4 (Group 1, number= 52) and moderate/high nausea scores ≥ 4 (Group 2, number= 50). Nearly half of the patients experienced early moderate/high PONV in our study. There was no difference between groups in demographic variables except motion sickness history (p=0.005) and thyroid disease (p=0.044) (Table 2). In Group 1, half of the patients (number= 26, 50%) were wearing acupressure bands, and the other half (number= 26, 50%) received ondansetron. In Group 2, 26 patients (52%) were wearing acupressure bands, and 24 patients (48%) received ondansetron.

**Table 2:** Demographic and perioperative variables of Group 1 and Group 2 Data are presented as the mean ± SD, absolute number (percentage), or median (interquartile range). *, Mann-Whitney U test; †, independent sample t-test; §, Chi-square test P values in bold represent statistically significant results (P < 0.05). BMI: Body mass index; ASA: American Society of Anesthesiologists; PONV: Post-operative nausea and vomiting; HADS: Hospital anxiety and depression scale; LOS: length of stay

Variables	Total (n= 102)	Group 1 (n= 52)	Group 2 (n= 50)	P value
Age, year	45 ± 10	45 ± 11	45 ± 10	0.989*
Height, cm	161 ± 6	161 ± 6	161 ± 6	0.690*
Weight, kg	73 ± 12	73 ± 11	73 ± 13	0.963^†^
BMI	28 ± 7	28 ± 5	29 ± 10	0.546*
Non-smokers, n	73 (71%)	36 (69%)	37 (74%)	0.593^§^
ASA, n				0.684^§^
I	11 (11%)	5 (10%)	6 (12%)	
II	81 (79%)	43 (82%)	38 (76%)	
III	10 (10%)	4 (8%)	6 (12%)	
Apfel score, n				0.076^§^
II	23 (22%)	15 (29%)	8 (16%)	
III	57 (56%)	30 (58%)	27 (54%)	
IV	22 (22%)	7 (13%)	15 (30%)	
Previous PONV, n	15 (15%)	9 (20%)	6 (15%)	0.711^§^
Motion sickness, n	28 (28%)	8 (15%)	20 (40%)	0.005^§^
Comorbidities, n				
HT	19 (19%)	13 (25%)	6 (12%)	0.092^§^
DM	13 (13%)	6 (11%)	7 (14%)	0.709^§^
Asthma	8 (8%)	4 (8%)	4 (8%)	0.954^§^
Thyroid	7 (7%)	1 (2%)	6 (12%)	0.044^§^
HADS				
Anxiety	7 ± 4	6 ± 4	7 ± 5	0.417*
Depression	6 ± 6	5 ± 3	6 ± 3	0.686*
Operation type, n				0.159^§^
Laparoscopy	29 (28%)	11 (21%)	18 (36%)	
Laparotomy	41 (40%)	21 (40%)	20 (40%)	
Vaginal	32 (32%)	20 (39%)	12 (24%)	
Intraoperative crystalloid fluid, ml	1250 (975- 2000)	1000 (800- 2000)	1500 (1075- 2000)	0,091*
Diuresis, ml	300 (200- 500)	300 (200- 530)	300 (200- 500)	0,612*
Heart rate, per min				
Before surgery (First recorded)	86 ± 17	86 ± 17	86 ± 12	0.805^†^
After surgery (Last recorded)	84 ± 16	84 ± 16	84 ± 13	0.887^†^
Variation	-2 ± 16	-2 ± 18	-2 ± 17	0.929^†^
Mean artery pressure, mmHg				
Before surgery (First recorded)	96 ± 13	96 ± 13	94 ± 10	0.484*
After surgery (Last recorded)	92 ± 14	92 ± 14	92 ± 13	0.965*
Variation	-4 ± 13	-4 ± 14	-2 ± 13	0.629*
Operation time, min	115 (75-160)	86 (30- 360)	120 (90- 166)	0.024*
Postoperative pain scores				
1^st^ hour	7.4 ± 2,9	6.7 ± 3.2	8.2 ± 2.4	0.012*
2^nd^ hour	6.3 ± 2.9	5.7 ± 3.1	7 ± 2.5	0.032*
6^th^ hour	5.2 ± 2.9	4.6 ± 3.1	5.8 ± 2.5	0.070*
12^th^ hour	3.8 ± 3.1	3.6 ± 3.1	4 ± 3.1	0.541*
24^th^ hour	2.9 ± 3	2.8 ± 3.1	3 ± 2.9	0.810*
Postoperative nausea scores				
1^st^ hour	3.6 ± 3.9	0.8 ± 1.7	6.4 ± 3.5	< 0.001*
2^nd^ hour	3.5 ± 4.1	0.3 ± 0.9	6.8 ± 3.4	< 0.001*
6^th^ hour	2.6 ± 3.8	1.5 ± 3.1	3.8 ± 4.1	0.001*
12^th^ hour	1 ± 2.6	0.5 ± 1.8	1.5 ± 3.1	0.048*
24^th^ hour	0.3 ± 1.3	0.1 ± 0.3	0.6 ± 1.9	0.045*
Postoperative vomiting scores				
1^st^ hour	2.4 ± 3.9	0.4 ± 1.7	4.4 ± 4.6	< 0.001*
2^nd^ hour	2.2 ± 3.9	0.3 ± 1.4	4.1 ± 4.6	< 0.001*
6^th^ hour	1.9 ± 3.8	1.2 ± 3.1	2.6 ± 4.2	0.070*
12^th^ hour	0.6 ± 2.2	0.5 ± 2	0.7 ± 2.5	0.914*
24^th^ hour	0.1 ± 1	0 ± 0.1	0.2 ± 0.8	0.695*
Anti-emetic treatment at ward, n	11 (10%)	0 (0%)	11 (22%)	< 0.001^§^
Removing bands, n	10 (19%)	2 (4%)	8 (16%)	0.039^§^
Hospital LOS, day	2.7 ± 0.9	2.7 ± 1.1	2.6 ± 0.8	0.695*

In perioperative variables; operation time (p=0.024), postoperative early pain scores, and vomiting scores were significantly different between groups (Table 2). Of all patients, 11 patients (10%) required an extra anti-emetic medication at the ward. Two patients in Group 1 and eight patients in Group 2 reported removing acupressure bands due to feeling uncomfortable. (p=0.039) Hospital LOS days were not significantly different between groups.

## Discussion

In this study, we first, aimed to assess the effect of PC6 point acupressure on PONV after gynecological surgeries compared to IV ondansetron. We did not find any significant difference between ondansetron and acupressure bands for early postoperative nausea. Twenty-four patients (48%) in Group O and 26 patients (52%) in Group B had moderate/high early PONV scores. Early PONV scores for Group O and Group B were not significantly different. Acupressure bands might be an alternative treatment to ondansetron without any side effects. This result was consistent with the literature [12].

In our study, patients recorded the highest PONV scores in the first and second hours after surgery. Supporting our results, Moore et al. showed higher PONV scores in the early period [13]. Our findings showed only one anti-emetic prevention, which was ondansetron or an acupressure band, was not adequate for early PONV. Of all patients, 11 (10%) required extra anti-emetic medication at the ward. Similar to our results, Moore et al. showed in their study that most of the patients with an Apfel score of 2 or 3, had received only one anti-emetic in the early phase [13]. Börjeson et al. performed a study about ward nurses’ experiences in PONV. Nurses declared that they felt a lack of knowledge of anti-emetic drugs for PONV treatment, that patients were not informed about PONV before the surgery, and that single rooms gave relief for PONV [14]. In our ward, two patients stay together in a room, and there is no private place for patients. 

Agarwal et al. and Yilmaz et al. assessed the effect of acupressure bands for PONV in different types of surgeries and showed no effect of acupressure bands on PONV [15,16]. Contrary to them, Kwon et al. showed the effect of acupressure bands for PONV on the first hour of the postoperative period [17], and Hofmann et al. found a significant effect up to 48 hours after surgery [6]. The variation of heart rates before and after surgery was significantly lower in Group O. Slowed heart rate is a side effect of Ondansetron. Operation time, intraoperative crystalloid fluid administration, and hospital LOS were significantly different between groups. These results might be influenced by each other without being related to acupressure or ondansetron. We can say that due to longer operation times, fluid administration was significantly higher in Group B. Also, there might be a relationship between operation time and hospital LOS.

Our other aim was to find the factors associated with early nausea. We compared low (<4) and moderate/high (≥4) mean nausea scores of the postoperative first and second hours. Motion sickness, thyroid disease, operation time, and postoperative first and second-hour pain scores were significantly different between groups. There were conflicting results about the history of motion sickness as a risk factor for PONV. While Roh et al. showed an association between motion sickness history and PONV [18], Zamoudio-Castilla et al. did not find an association [19]. Contrary to our results, Roh et al. also showed that preoperative high anxiety affected PONV [18].

In the current study, operation time was significantly different between Group O and Group B, but this might be a confounding factor for the first comparison. In the second comparison, which assessed factors associated with early postoperative nausea scores, the operation time was also significantly different. The effect of operation time on PONV has been confirmed by many experiments [17-19]. It was reported that every half-hour increase in the operation time increased the risk of PONV by 59%. [20]. Qian et al. showed that an operation time of more than one hour, which was two hours in Group 2 in our study, affected PONV. They also showed an association of operation type with PONV, of which we did not find any association [19].

The current study confirmed the findings of clear support for the association between postoperative pain and PONV [18]. Pain and vomiting centers are located in the thalamus. Stimulation of a particular site may cause an interaction between neighboring nuclei and thus stimulate other nuclei. On the contrary, Stadler et al. did not find an association between postoperative pain scores and PONV [21].

## Conclusions

The effect of PC6 point acupressure on early PONV compared to IV ondansetron was similar after gynecological surgeries. However, using only one anti-emetic treatment did not adequately relieve early PONV. Of all patients, 11 (10%) required extra anti-emetic medication at the ward. Operation time, intraoperative crystalloid fluid administration, and hospital LOS were significantly different between Group O and Group B. These results might be influenced by each other without being related to acupressure or ondansetron. We can say that due to longer operation times, fluid administration was significantly higher in Group B. There might be a relationship between operation time and hospital LOS.

In our study, half of the patients experienced early moderate/high PONV. Motion sickness history, thyroid disease, operation time, and early postoperative pain scores were found to be the factors associated with early PONV.
